# Practice of sedation and analgesia in German intensive care units: results of a national survey

**DOI:** 10.1186/cc3035

**Published:** 2005-01-26

**Authors:** Jörg Martin, Axel Parsch, Martin Franck, Klaus D Wernecke, Matthias Fischer, Claudia Spies

**Affiliations:** 1 Senior physican, Department of Anesthesiology, Intensive Care Medicine and Pain Therapy, Hospital am Eichert, Göppingen, Germany; 2 Assistant physician, Department of Anesthesiology, Intensive Care Medicine and Pain Therapy, Hospital am Eichert, Göppingen, Germany; 3 Assistant physician, Department of Anesthesiology and Intensive Care Medicine, University Hospital Charité, Berlin, Germany; 4 Chairman, Institute of Medical Biometrics, University Hospital Charité, Berlin, Germany; 5 Chairman, Department of Anesthesiology, Intensive Care Medicine and Pain Therapy, Hospital am Eichert, Göppingen, Germany; 6 Professor of Anesthesiology and Chairman, Department of Anesthesiology and Intensive Care Medicine, University Hospital Charité, Berlin, Germany

## Abstract

**Introduction:**

Sedation and analgesia are provided by using different agents and techniques in different countries. The goal is to achieve early spontaneous breathing and to obtain an awake and cooperative pain-free patient. It was the aim of this study to conduct a survey of the agents and techniques used for analgesia and sedation in intensive care units in Germany.

**Methods:**

A survey was sent by mail to 261 hospitals in Germany. The anesthesiologists running the intensive care unit were asked to fill in the structured questionnaire about their use of sedation and analgesia.

**Results:**

A total of 220 (84%) questionnaires were completed and returned. The RAMSAY sedation scale was used in 8% of the hospitals. A written policy was available in 21% of hospitals. For short-term sedation in most hospitals, propofol was used in combination with sufentanil or fentanyl. For long-term sedation, midazolam/fentanyl was preferred. Clonidine was a common part of up to two-thirds of the regimens. Epidural analgesia was used in up to 68%. Neuromuscular blocking agents were no longer used.

**Conclusion:**

In contrast to the US 'Clinical practice guidelines for the sustained use of sedatives and analgesics in the critically ill adult', our survey showed that in Germany different agents, and frequently neuroaxial techniques, were used.

## Introduction

Critical care therapies such as ventilation, invasive procedures or other measures inducing pain or stress require analgesia and sedation of the patient. Adequate analgesia and sedation is supposed to prevent stress-induced reactions such as hypermetabolism, sodium and water retention, hypertension, tachycardia and altered wound healing [1-3] and to optimize patient comfort. Whipple and colleagues [4] pointed out that 70% of the patients in an intensive care unit (ICU) indicate pain as the worst recollection, although 70–90% of the nurses and physicians taking care of them claimed their patients to be pain free. If sedation is too deep it can have negative side effects [5-7] such as increased risk of pneumonia, venous thrombosis, bowel motility problems, hypotension and a prolonged stay in the ICU, resulting in increased costs [8-10]. The requirements for ideal analgesia and sedation are the ability to sedate the patient deeply for necessary procedures, but with medication of short duration so that the patient can be quickly responsive and cooperative [11].

Goal-oriented sedation [5,6,12,13] complies with the establishment of a modern ventilation regimen to allow early spontaneous breathing [14]. This is shown in the use of short-acting agents for analgesia and sedation, as was demonstrated in surveys from the UK [15] and Denmark [16].

Soliman and colleagues [17] conducted a survey in 16 European countries about the current practice of sedation. They found distinct differences between countries in the practice of analgesia and sedation: 75% of ventilated patients in the UK received medication for analgesia and sedation continuously, whereas only 30% of Italian ventilated patients did so. The most commonly used medication for continuous sedation in Europe is propofol and midazolam, whereas in the USA [18] midazolam and propofol is administered as the preferred medication for short-term sedation and lorazepam for long-term sedation. Analgesic agents differ broadly between countries, and neuroaxial techniques are not often reported [15-18].

The goal of the present survey was to ask for the current practice of analgesia and sedation in German ICUs.

## Methods

### Data collection

From an address database of the German Society for Anesthesiology and Intensive Care Medicine (Deutsche Gesellschaft für Anästhesiologie und Intensivmedizin [DGAI]) with a total of 808 addresses of ICUs (45 university hospitals and 763 general hospitals) a simple random sample of one-third of the addresses (254 general hospitals and 15 university ICUs) were selected and written to. For eight hospitals the letter was undeliverable. It was written up to four times to the hospitals during May 2002 to October 2002. The return rate was 84% (220 of 261). All data were included in the analysis. At this return rate the 'non-responder bias' can be neglected [19]. The hospitals included in the analysis were 206 general hospitals and 14 university hospitals. Numbers of hospital beds varied between less than 300 beds in 71 hospitals, 300–499 in 88 hospitals, 500–1000 in 43 hospitals, and more than 1000 beds in 14 hospitals; 2 hospitals did not answer. The questionnaire itself is provided in Additional file 1.

### Duration of sedation

The periods of sedation were clustered in accordance with the American guidelines [18] into the following groups: duration of sedation less than 24 hours, 24–72 hours and more than 72 hours. In addition we asked about the duration of sedation during weaning from ventilation.

### Statistics

The data were collected in a Microsoft Access 97 database and analyzed with the programs Microsoft Excel 9.0 and SPSS for Windows (SPSS Inc., version 10.07). Univariate statistitical analyses were calculated depending on the scaling of the data with the Mann–Whitney *U*-test or the χ^2 ^test. If multiple tests in multigroup comparison were necessary, we used the Bonferroni–Holm sequential rejective multiple test procedure [20].

Significant two-sided differences were defined as *P *< 0.05.

## Results

### General data

General hospitals were equipped with a median of 9 intensive care beds (university hospitals 14), the median number of patients in the ICU was 930 (university hospitals 1481) and the resulting median nursing care days per year were 2565 (university hospitals 4950).

### Procedure instructions for analgesia and sedation

Oral procedure instructions (departmental common consensus) existed in 43% of the hospitals.

A standard operating procedure set out in writing existed in 21% of the hospitals.

### Use of sedation scales

Sedation monitoring was done by 30% of the hospitals. It was noticeable that only 8% of the hospitals provided data for the question about which sedation scale was used. The Ramsay scale [21] was named exclusively as the sedation scale used.

### Medication costs

Sixty-two percent of all hospitals answered 'yes' when asked about considering costs in their choice of medication. The analysis showed that there were no significant differences in the choice of agents compared with the hospitals that said 'no' to this question (*P *= 0.758).

### Choice of agents depending on the duration of sedation

Ninety-two percent of the hospitals stated that they selected the medication depending on the expected duration of analgesia and sedation.

### Day–night rhythm

Eighty-one percent of the hospitals tried to maintain a day–night rhythm in their patients.

### Neuromuscular blockade

Neuromuscular blockade no longer had a role in ICUs run by anesthesiologists.

### Withdrawal/transitional syndrome

Questioned about the frequency of withdrawal/transitional syndrome, the hospitals stated an average rate of 20%.

### Sedative agents

For sedation up to 24 hours, propofol was used significantly more often (81%) as a continuous agent than midazolam (45%, *P *< 0.05). For sedation between 24 and 72 hours, midazolam was used significantly more often (77%) than propofol (56%). For sedation longer than 72 hours, all hospitals preferred midazolam (90%) as a sedative and only 25% of the hospitals were using propofol. During weaning, 92% of the hospitals used propofol for sedation, and 34% used midazolam.

### Analgesic agents

For analgesia up to 24 hours, sufentanil (35%) and fentanyl (40%) were used most often. There was no significant difference in the use of these two agents. Thirty-eight percent of the hospitals saw an indication for the use of piritramid, non-steroidal anti-inflammatory drugs (NSAIDs) were used by 27%, morphine was used by 9%, alfentanil by 2%, remifentanil by 6%, and hydromorphone was not an option in our questionnaire. It was not possible to derive from the data how often opioids and NSAIDs were used as additional agents for analgesia or as alternative drugs. For analgesic agents between 24 and 72 hours, fentanyl (55%) and sufentanil (53%) were used by most of the hospitals. Morphine was used by 5%, piritramid by 16%, alfentanil by 2% and remifentanil by 2%. Twelve percent of the hospitals saw an indication for the use of NSAIDs. For analgesia of more than 72 hours, fentanyl was used in 64% of hospitals, significantly more often than sufentanil (44%) was used. The use of piritramid occurred in 9% of all hospitals, morphine was used by 7%, alfentanil by 1% and remifentanil by 1%. Ten percent of all hospitals were using NSAIDs. For analgesia during weaning from ventilation, 39% of the hospitals used fentanyl and 42% used sufentanil. No significant difference was shown between the usage of these two agents. In all hospitals piritramid was used by 25%, morphine by 9%, alfentanil by 2%, remifentanil by 6% and NSAIDs by 14% in this phase.

### Adjuvant techniques for analgesia and sedation

Clonidine was used by 35% of the hospitals for sedation of less than 24 hours; 7% of the respondent hospitals decided on ketamine (S). Haloperidol was not a selectable option in our questionnaire. For sedation between 24 and 72 hours, clonidine was used in 48% of the hospitals, and ketamine (S) by 20%. For sedation longer than 72 hours, clonidine was used by 56% of all the hospitals, and 19% were using ketamine (S). During weaning from ventilation, the α2 agonist clonidine was used by 62% of all hospitals, and 5% used ketamine (S) in this phase.

### Regional anesthetic techniques

A central neuroaxial block with an epidural catheter was used by 68% of all hospitals as a routine for analgesia up to 24 hours. Peripheral blocks were used by 15% of all hospitals. As a regional anesthetic technique for analgesia between 24 and 72 hours, epidural analgesia was used by 60% of all hospitals, and peripheral blocks were used by 13%. For analgesia and sedation longer than 72 hours, epidural analgesia was used by 46% of all hospitals, and peripheral blocks were used by 11%. Epidural analgesia was used by 43% of all hospitals during ventilator weaning, and peripheral blocks were used by 7% of all hospitals.

The use of the different agents and techniques is summarized in Table 1.

## Discussion

The most important result is the use of different agents according to the expected length of analgesia and sedation. In the American guidelines [18] for short-term sedation only propofol is recommended, and for long-term sedation midazolam and lorazepam are recommended. In our survey the most commonly used agent for sedation up to 24 hours and during weaning from ventilation was propofol. Midazolam was used mainly for sedation longer than 72 hours. Lorazepam was not used by any department. This is due mainly to handling (2 mg ampoule) and costs, because lorazepam is one of the more expensive agents in Germany for sedation.

Whereas the American guidelines recommend fentanyl, hydromorphone and morphine for analgesia in all phases [18], in our survey fentanyl and sufentanil were used most often for analgesia for up to 24 hours, between 24 and 72 hours and for weaning from ventilation. In addition, NSAIDs were used preferentially in short-term sedation (less than 24 hours). For longer than 72 hours, fentanyl was preferred for analgesia. Whereas in the British survey [15] from the year 2000 alfentanil was very often used, this opioid did not have a role in German ICUs. In the Danish survey the preferred drugs for analgesia were morphine (94%), fentanyl (76%) and sufentanil (43%) [16]. Our survey shows that morphine did not have a major role in analgesia and sedation in German ICUs. Specifically in Germany, piritramide is a frequently used agent for postoperative analgesia. The reason may be that in Germany some anesthesiologists claim to achieve a lower incidence of nausea and vomiting with piritramid than with morphine [22].

Noticeable was the widespread use of central neuroaxial techniques in analgesia for up to 24 hours. Brodner and colleagues [23] and Beattie and colleagues [24] showed that the perioperative use of epidural analgesia leads to a shortened length of stay in the ICU and also a decrease in cardiac events.

Clonidine as an adjuvant for sedation was used in our survey in a high percentage in all phases, whereas haloperidol, which is recommended in the American guidelines, was not a selectable option in our questionnaire. Most often clonidine was used in the phases longer than 72 hours and during weaning from ventilation. A reasonable use (with regard to time of ventilation and ICU stay) of this agent during weaning was shown by Walz and colleagues [25]. Bohrer and colleagues showed [26] that with clonidine the requirements for opioids and sedation may be reduced.

Ketamine (S) was preferred as an adjuvant in the phases of sedation longer than 24 hours. There have been few studies for long-term sedation with ketamine, as Ostermann and colleagues [11] showed in their review. One of the reasons for the use of ketamine is the lower negative influence on bowel motility than with opioids [27].

In our survey 43% of the hospitals stated that they had have established an oral policy for analgesia and sedation. A procedure in writing was used in 21%. In the survey by Murdoch and colleagues [15] 43% of the British ICUs stated that they had procedures in writing for analgesia and sedation, and 51% had a defined oral policy. In addition, in the 1987 survey of British ICUs by Bion and colleagues [28], 40% stated that they had established a formal procedure. In other surveys it was shown that with the use of standard operating procedures a decrease in the durations of sedation and ventilation, and with this a reduction of costs, is possible [29,30]. Mascia and colleagues [31] showed that the use of written standard operating procedures decreases the duration of ventilation, the length of stay in the ICU and the overall hospital stay.

A sedation scale for the monitoring of analgesia and sedation was used by 31% of the hospitals questioned; 8% stated that they used the Ramsay sedation scale [21] for monitoring sedation. In the survey by Soliman and colleagues [17], 49% of the German hospitals answered that they used the Ramsay sedation scale [19]. In English hospitals in the survey by Murdoch and colleagues [15], 60% were using a sedation scale. In the survey of Danish ICUs [16] from the year 1996/1997, 16% of the hospitals answered that they were using a sedation scale.

More recent studies showed that close monitoring with the help of a scoring system can lead to a decrease in the length of ICU stay and in the length of hospital stay [32].

Nearly all hospitals in our survey stated that they paid attention to cost in their choice of medication. However, the survey showed that there were no significant differences in the use of medications between the hospitals that answered yes and those that answered no. Murdoch and colleagues [15] came to the same conclusion in their survey of English ICUs. More expensive agents may be useful with regard to overall costs because the length of stay in the ICU may be reduced, as was shown by Barrientos-Vega and colleagues [33] and Dahaba and colleagues [34].

Questioned on whether the expected length of sedation had a role in selecting the medication, 92% of the hospitals agreed. The analysis showed that for short-term sedation agents were also used that had a long context-sensitive half-time (fentanyl 45%, midazolam 40%) [35]. Nearly all ICUs tried to maintain a day–night rhythm, although only few studies exist [36,37] that have shown advantages of it for the patients. The Danish survey [16] yielded almost the same results.

In our survey the use of neuromuscular blocking agents had almost disappeared, confirming the results of the European [17], British [15] and Danish [16] surveys of the routine use of neuromuscular blocking agents in intensive care medicine.

The incidence of withdrawal in long-term sedation is 60–80% [38,39]. In our survey, values between 20% and 25% were stated, which is explained by the fact that all patients, even short-term patients, were included.

In our survey the return rate was 84%. Christensen and colleagues [16] in Denmark and Murdoch and colleagues [15] in the UK achieved similar return rates (92.5% and 79%, respectively). In a pan-European questionnaire about the practice of analgesia and sedation by Soliman and colleagues [17] the return rate was 20%.

One of the problems of this survey was the limitation to ICUs run by the department of anesthesiology. We do not have data on whether the patients were mainly postoperative and trauma patients, or whether the ICUs also routinely took care of patients from the department of internal medicine. Hack and colleagues showed in their survey [40] that the most of the interdisciplinary ICUs in general hospitals in Germany are run by the department of anesthesiology [40].

## Conclusion

In German anesthesiological ICUs the main short-acting agent used for sedation was propofol, and the benzodiazepine midazolam was used for long-term sedation. For analgesia the opioids fentanyl and sufentanil were used. A very large proportion of hospitals used epidural analgesia. In addition, clonidine was very often used as an adjuvant agent. Only a small proportion of hospitals had established a sedation protocol in writing or a scoring system for the monitoring of analgesia and sedation, although numerous publications showed that the consistent use of these methods can lead to a decrease in ventilator time and length of ICU stay [29-31].

## Key messages

• 	Very little sedation monitoring is used in German intensive care units.

• 	Only few intensive care units use guidelines for analgesia and sedation.

• 	Neuroaxial techniques are commonly applied.

## Abbreviations

NSAIDs = non-steroidal anti-inflammatory drugs; ICU = intensive care unit.

## Competing interests

The author(s) declare that they have no competing interests.

## Authors' contributions

JM made substantial contributions to the conception, design, analysis and interpretation of data. AP performed the acquisition, analysis and interpretation of data. MF was involved in drafting the article and revising it critically for important intellectual content. KDW participated in the design of the study and performed the statistical analysis. MF participated in the design and coordination and helped to draft the manuscript. CS made substantial contributions to the conception, design, analysis and interpretation of data. All authors read and approved the final manuscript.

## Supplementary Material

Additional File 1 A pdf file containing the questionnaire. Click here for file

## Figures and Tables

**Figure 1:**
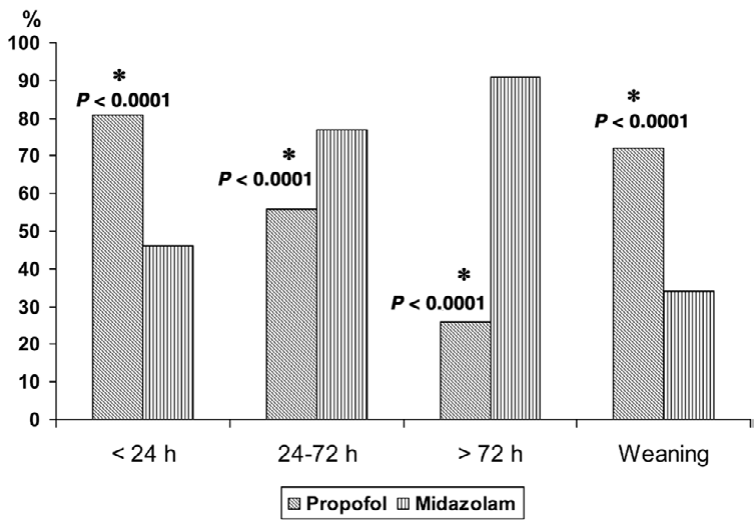
The most commonly used sedative agents for the different sedation periods. *Differences between midazolam and propofol in a phase (χ^2 ^test, *P *< 0.05). The values were tested with the χ^2 ^test (*P *< 0.05) and multiple differences with the Bonferroni–Holm multiple test procedure. (Propofol: less than 24 hours versus 24–72 hours versus more than 72 hours versus weaning, all *P *< 0.0001. Midazolam: less than 24 hours versus 24–72 hours versus more than 72 hours versus weaning, all *P *< 0.0001.)

**Figure 2:**
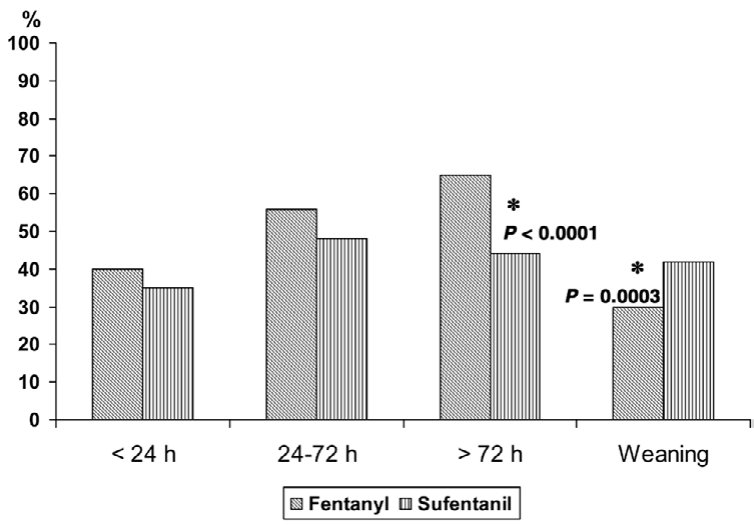
The most commonly used analgesic agents during the different sedation periods. *Differences between fentanyl and sufentanil in a phase (χ^2 ^test, *P *< 0.05). The values were tested with the χ^2 ^test (*P *< 0.05) and multiple differences with the Bonferroni–Holm multiple test procedure. (Fentanyl: less than 24 hours versus 24–72 hours versus more than 72 hours versus weaning, all *P *< 0.01. Sufentanil: less than 24 hours versus 24–72 hours; less than 24 hours versus more than 72 hours; 24–72 hours versus more than 72 hours and 24–72 hours versus weaning, all *P *= 0.01; less than 24 hours versus weaning, *P *= 0.04; more than 72 hours versus weaning, *P *= 0.55.)

**Figure 3:**
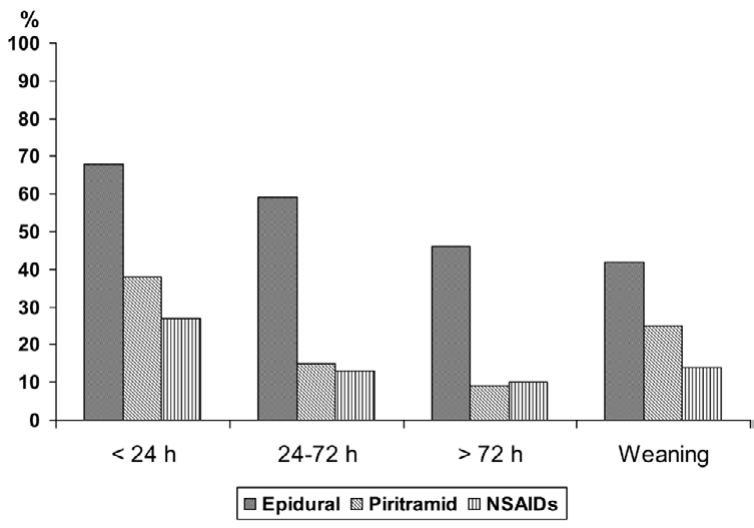
Epidural analgesia, piritramid and NSAIDs in the different phases of analgesia and sedation. The values were tested with the χ^2 ^test (*P *< 0.05) and multiple differences with the Bonferroni–Holm multiple test procedure. NSAIDs, non-steroidal anti-inflammatory drugs. (Epidural: less than 24 hours versus more than 72 hours; less than 24 hours versus weaning; 24–72 hours versus more than 72 hours and 24–72 hours versus weaning, all *P *< 0.01; less than 24 hours versus 24–72 hours, *P *= 0.015; more than 72 hours versus weaning, *P *= 0.37. Piritramid: less than 24 hours versus 24–72 hours versus more than 72 hours versus weaning, all *P *< 0.01. NSAIDs: less than 24 hours versus 24–72 hours, less than 24 hours versus more than 72 hours and less than 24 hours versus weaning, all *P *< 0.0001; 24–72 hours versus more than 72 hours, *P *= 0.14; 24–72 hours versus weaning, *P *= 0.67; more than 72 hours versus weaning, *P *= 0.087.)

**Figure 4:**
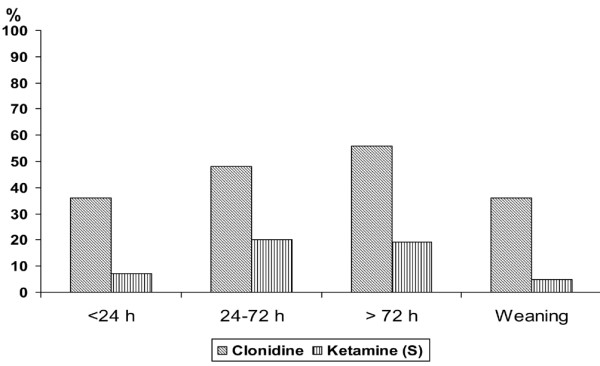
The most commonly used adjunct techniques in the different phases of analgesia and sedation. The values were tested with the χ^2 ^test (*P *< 0.05) and multiple differences with the Bonferroni–Holm multiple test procedure. (Clonidine: less than 24 hours versus more than 72 hours, less than 24 hours versus weaning and 24–72 hours versus weaning, all *P *< 0.01; 24 hours versus 24–72 hours, *P *= 0.23; 24–72 hours versus more than 72 hours, *P *< 0.017; more than 72 hours versus weaning, *P *= 0.067. Ketamine (S): less than 24 hours versus 24–72 hours, less than 24 hours versus more than 72 hours, 24–72 hours versus weaning and more than 72 hours versus weaning, all *P *< 0.0001; less than 24 hours versus weaning, *P *= 0.22; 24–72 hours versus more than 72 hours, *P *= 0.087.)

**Table 1:** Comparison of the used agents and techniques for analgesia and sedation

Agent/technique	Percentage (95% confidence interval)			
	
	< 24 h	24–72 h	>72 h	Weaning
Midazolam	45.9 (36.1–55.6)	77.3 (71.0–83.6)	90.5 (86.4–94.5)	34.1 (23.4–44.8)
Diazepam	0.0	2.7 (- 10.3–15.8)	3.2 (- 9.8–16.2)	2.3 (- 10.8–15.3)
Propofol	81.4 (75.7–87.1)	55.9 (47.2–64.7)	26.4 (15.0–37.7)	72.3 (65.3–79.3)
Methohexital	1.4 (- 11.8–14.5)	2.7 (- 10.3–15.8)	4.1 (- 8.9–17.0)	2.3 (- 10.8–15.3)
GHBA	4.5 (8.4–17.5)	7.7 (- 5.0–20.4)	10.9 (- 1.5–23.4)	9.6 (- 3.0–22.1)
Remifentanil	5.9 (- 6.9–18.7)	2.3 (- 10.8–15.3)	1.4 (- 11.8–14.5)	5.9 (- 6.9–18.7)
Alfentanil	1.8 (- 11.3–14.9)	2.3 (- 10.8–15.3)	0.9 (- 12.3–14.1)	1.8 (- 11.3–14.9)
Fentanyl	40.0 (29.8–50.3)	55.9 (47.1–64.7)	65.0 (57.2–72.8)	30.0 (19.0–41.0)
Sufentanil	35.0 (24.4–45.7)	47.7 (38.2–57.3)	43.6 (33.7–53.5)	41.8 (31.8–51.9)
Piritramid	38.2 (27.8–48.6)	15.5 (3.3–27.6)	9.1 (- 3.5–21.7)	25.5 (14.1–36.9)
Morphine	8.6 (- 4.0–21.3)	4.5 (- 8.4–17.5)	7.3 (- 5.5–20.0)	8.6 (- 4.0–21.3)
PCA	25.5 (14.0–36.9)	15.5 (3.3–27.6)	9.5 (- 3.0–22.1)	12.3 (- 0.1–24.7)
Ketamine (S)	6.8 (- 5.9–19.6)	20.0 (8.2–31.8)	19.1 (13.6–36.4)	5.0 (- 7.9–17.9)
Clonidine	35.9 (25.3–46.5)	48.2 (38.7–57.7)	56.4 (47.7–65.1)	62.7 (54.6–70.8)
NSAIDs	26.8 (15.6–38.2)	13.2 (0.8–25.5)	10.5 (- 2.1–23.0)	14.1 (1.8–26.3)
NMBAs	3.6 (- 9.3–16.6)	0.0	0.0	0.0
PNB	15.5 (3.3–27.6)	12.7 (0.4–25.1)	10.0 (- 2.5–22.5)	7.7 (- 5.0–20.4)
PCEA	7.3 (- 5.5–20.0)	5.9 (- 6.9–18.7)	4.1 (- 8.9–17.0)	4.6 (- 8.4–17.5)
Epidural	68.2 (60.8–75.7)	59.1 (50.7–67.6)	45.9 (36.2–55.6)	42.3 (32.2–52.3)

GHBA, γ-hydroxybutyric acid; NMBAs, neuromuscular blocking agents; NSAIDs, non-steroidal anti-inflammatory drugs; PCA, patient-controlled analgesia; PCEA, patient-controlled epidural analgesia; PNB, peripheral nerve block.
