# Supplementary material for: Practice of sedation and analgesia in German intensive care units: results of a national survey
Source: Crit Care. 2005 Jan 26;9(2):R117–23. doi: 10.1186/cc3035 (PMC1175921; doi:10.1186/cc3035)
Supplement: Additional File 1 — A pdf file containing the questionnaire. [file cc3035-S1.pdf]

Additional file

The questionnaire

Questions about the hospital structure

1. Number of beds of the hospital
2. Structure of the intensive care unit

a) Number of beds ________ beds
b) Number of patients ________ patients per year
c) Number of patient care days ________ patient care days per year
d) Portion of ventilated patients ________ %
e) Number of anesthetics per year ________

Questions on the practice of sedation and analgesia

1. Do you use routinely a sedation scale (e.g. Ramsay)?
2. Which sedation scale do you use?
3. Do you use routinely neuromuscular blockade for sedated and ventilated patients?
4. Do you attempt to create a day-night rhythm?
5. Do you have put down a sedation management in writing?
6. On how many percent of patients with analgesia and sedation do you experience a transitional syndrome/delir (estimate)?
7. Do costs play a role in the selection of agents for sedation?
8. Does the expected duration for sedation play a role in the selection of agents for sedation?
9. Which medication or technique do you use for analgesia and sedation up to 24 h, 24 to 72 h, > 72 h and for weaning?

**Sedation**		**Analgesia**		**Miscellaneous**	
**Medication**		**Medication**		**Medication**	
Midazolam	❑	Morphine	❑	Epidural	❑
Propofol	❑	Fentanyl	❑	Peripheral block	❑
Methohexital	❑	Sufentanil	❑	Clonidine	❑
Gamma-hydroxy-butyric acid	❑	Alfentanil	❑	PCA	❑
Diazepam	❑	Remifentanil	❑	PCEA	❑
Lorazepam	❑	Piritramid	❑	NMB	❑
		Ketamine (S)	❑		
Miscellaneous	❑	NSAIDs	❑	Miscellaneous	❑
			❑		

The above table was used four times for every sedation period respectively. Abbreviations: NSAIDs = Non-steroidal anti-inflammatory drugs, PCA = patient controlled analgesia, PCEA = patient controlled epidural analgesia, NMB = neuromuscular blocking agents. Multiple choices were possible
